# Birth Weight Ratio as an Alternative to Birth Weight Percentile to Express Infant Weight in Research and Clinical Practice: A Nationwide Cohort Study

**DOI:** 10.1155/2014/749476

**Published:** 2014-08-13

**Authors:** Bart Jan Voskamp, Brenda M. Kazemier, Ewoud Schuit, Ben Willem J. Mol, Maarten Buimer, Eva Pajkrt, Wessel Ganzevoort

**Affiliations:** ^1^Department of Obstetrics and Gynecology, Room H4-232, Meibergdreef 9, Academic Medical Center, 1105 AZ Amsterdam, The Netherlands; ^2^Julius Center for Health Sciences and Primary Care, University Medical Center Utrecht, Universiteitsweg 100, 3584 CG Utrecht, The Netherlands; ^3^The Robinson Institute, School of Paediatrics and Reproductive Health, University of Adelaide, Adelaide, SA 5005, Australia; ^4^Department of Obstetrics and Gynecology, Skaraborgs Sjukhus, Skövde, 541 85 Västra Götaland, Sweden

## Abstract

*Objective*. To compare birth weight ratio and birth weight percentile to express infant weight when assessing pregnancy outcome. *Study Design*. We performed a national cohort study. Birth weight ratio was calculated as the observed birth weight divided by the median birth weight for gestational age. The discriminative ability of birth weight ratio and birth weight percentile to identify infants at risk of perinatal death (fetal death and neonatal death) or adverse pregnancy outcome (perinatal death + severe neonatal morbidity) was compared using the area under the curve. Outcomes were expressed stratified by gestational age at delivery separate for birth weight ratio and birth weight percentile. *Results*. We studied 1,299,244 pregnant women, with an overall perinatal death rate of 0.62%. Birth weight ratio and birth weight percentile have equivalent overall discriminative performance for perinatal death and adverse perinatal outcome. In late preterm infants (33^+0^–36^+6^ weeks), birth weight ratio has better discriminative ability than birth weight percentile for perinatal death (0.68 versus 0.63, *P*  0.01) or adverse pregnancy outcome (0.67 versus 0.60, *P* < 0.001). *Conclusion*. Birth weight ratio is a potentially valuable instrument to identify infants at risk of perinatal death and adverse pregnancy outcome and provides several advantages for use in research and clinical practice. Moreover, it allows comparison of groups with different average birth weights.

## 1. Introduction

Gestational age at delivery and birth weight are considered important predictors of adverse pregnancy outcome [1, 2]. Accurate assessment of fetal growth in relation to gestational age is therefore an important tool for risk assessment in antenatal care.

Fetal growth is usually expressed in percentiles. Birth weight percentile curves are calculated from cross-sectional data of newborns [3]. Thus, birth weight percentiles (BWpercentiles) indicate the value (e.g., 10%) below which a certain percentage of the observations in a group of newborns (10%) can be found. BWpercentiles are often dichotomized, and small for gestational age (SGA) is commonly defined as birth weight below the 10th, 5th, or 2.3th percentile for gestational age in a population-specific reference growth curve [4, 5]. The BWpercentile tells us if an infant belongs to a certain part of the percentile distribution but does not contain any information about the absolute deviation of infant weight from the median birth weight for gestation. As a result, percentiles do not allow comparison of growth between groups with different growth characteristics (e.g., different sexes or ethnicities). Moreover, at the tails of the normal distribution (e.g., at the 2nd percentile), a percentile contains a much wider range of absolute birth weights than close to the median (e.g., at the 50th percentile). Consequently, the use of percentiles and their dichotomization may lead to loss of information that may be useful for patient care and parental counseling.

Birth weight ratio (BWratio) is an alternative method to express growth of an individual with respect to the median. It is defined as the ratio of observed birth weight divided by the median birth weight of the population-specific reference growth curve. Values above 1 indicate “larger for gestational age than the median” and values below 1 indicate “smaller for gestational age than the median.” It may offer a solution to the limitations associated with BWpercentiles.

Our objective was to compare BWratio and BWpercentile to express infant growth when assessing pregnancy outcome.

## 2. Materials and Methods

### 2.1. Dataset

This study was performed using a nationwide cohort using data from The Netherlands Perinatal Registry (PRN). The PRN consists of population-based data on pregnancies, deliveries, neonatal characteristics, and readmissions until 28 days after birth. The PRN database is obtained by a validated linkage of three different registries, the midwifery registry, the obstetrics registry, and the neonatology registry of hospital admissions of newborn neonates [6, 7]. Records are entered in the PRN registry at the child's level. The coverage of the PRN registry is approximately 96% of all deliveries in The Netherlands. It contains pregnancies of ≥22 weeks' gestation and a birth weight of ≥500 g and is used primarily for an annual assessment of the quality indicators of obstetric care.

### 2.2. Ethical Approval

The data in the perinatal registry are anonymous; therefore ethical approval was not needed. The Dutch Perinatal Registry gave their approval to use their data for this study (approval number 13.72).

### 2.3. Inclusion and Exclusion Criteria

We included all white women who delivered a singleton between 25^+0^ and 42^+6^ weeks gestation in The Netherlands between January 1, 1999, and December 31, 2007. All cases with congenital anomalies were excluded [8].

### 2.4. Outcome Measures

Outcome measures were perinatal death and a composite of perinatal death and neonatal morbidity. Perinatal death was defined as the sum of intrauterine fetal death (diagnosed after 25^+0^ weeks GA) and neonatal death (until 28 days after birth). The composite of adverse pregnancy outcome consisted of perinatal death, respiratory distress syndrome (RDS), sepsis, necrotizing enterocolitis (NEC), meconium aspiration, and intraventricular hemorrhage (IVH) within the first month of birth. If an infant suffered from neonatal morbidity and died within 28 days after birth, it was only considered as perinatal death in the analyses.

The Dutch reference curves for birth weight by gestational age stratified for parity, sex, and ethnic background were used [9]. Pregnancy dating was performed using last menstrual period (LMP) or by ultrasound measurements before 20 weeks of gestation (crown-rump-length (CRL) or head-circumference (HC) measurement).

We defined SGA as birth weight below the 10th or 5th percentile for gestation. To obtain the best possible comparability with SGA, low BWratio cut-off values of 0.85 and 0.80 were chosen such that (after rounding them to the closest 0.05 value) they resulted in equally large groups of low BWratio infants in the whole population as with the 10th and 5th birth weight percentiles.

We defined LGA as birth weight above the 90th or 95th percentile for gestation. To obtain the best possible comparability with LGA, high BWratio cut-off values 1.25 and 1.30 were chosen such that (after rounding them to the closest 0.05 value) they resulted in groups of high BWratio infants in the whole population that corresponds best with the 90th and 95th birth weight percentiles.

### 2.5. Population Characteristic and Clinical Characteristics

We registered demographic and obstetric characteristics including maternal age, parity, and socioeconomic status (SES) [10]. Parity was categorized into 0 (first birth), 1 (second birth), and 2+ (third or higher birth).

### 2.6. Statistics

Baseline characteristics were described and presented as means with standard deviations (SD), median with range, or percentages as appropriate.

We tested for interaction between BWratio and GA at delivery as well as BWpercentile and GA at delivery. These tests were performed separately for the two outcome measures. If statistically significant (*P* < 0.05), analyses were performed stratified for gestational age at delivery in four categories according to the WHO criteria, extremely preterm (24^+0^–27^+6^ weeks' gestation), very preterm (28^+0^–32^+6^ weeks' gestation), moderate to late preterm (33^+0^–36^+6^ weeks' gestation), and term delivery (37^+0^–42^+6^ weeks' gestation) [11].

We plotted distributions of perinatal death and adverse pregnancy outcome for BWratio and BWpercentile. In addition distributions of perinatal death and adverse pregnancy outcome stratified for gestational age at delivery for BWratio and BWpercentile were plotted.

We also calculated—separate for BWratio and BWpercentile and four strata of gestational age at birth—the population-attributable risk (PAR) of abnormal fetal growth for perinatal death and adverse pregnancy outcome. PAR was based on the prevalence (*P*) of abnormal growth and the relative risk (RR) of perinatal death and adverse pregnancy outcome in abnormally grown (low BWratio, SGA) and normally grown infants: PAR% = [*P*∗(RR − 1)/(*P*∗(RR − 1) + 1)]∗100 [12].

Finally, receiver operator characteristics (ROC) curves were constructed for the whole cohort and for abnormally grown infants only, to compare discriminative ability of birth weight ratio and birth weight percentile for our outcome measures (perinatal death and adverse pregnancy outcome) in the four gestational categories. All statistical tests were 2-sided; a probability value of 0.05 was chosen as the threshold for statistical significance.

The data were analyzed with the SAS statistical software package (version 9.2; SAS Institute Inc., Cary, NC).

## 3. Results

From January 1, 1999 until December 31, 2007 a total of 1,636,565 pregnancies were registered in the PRN database. We excluded cases that were nonwhite (*n* = 258,908 (15.82%)), multiple pregnancies (*n* = 63,857 (3.90%)), infants with congenital anomalies (*n* = 22,043 (1.35%)), and infants born before 25^+0^ weeks or after 42^+6^ weeks GA (*n* = 6,967 (0.43%)). After application of the inclusion and exclusion criteria the study population consisted of 1,299,244 pregnancies. Baseline characteristics of the population are shown in Table 1.

### 3.1. Distribution of Cases

**Figure 1:**
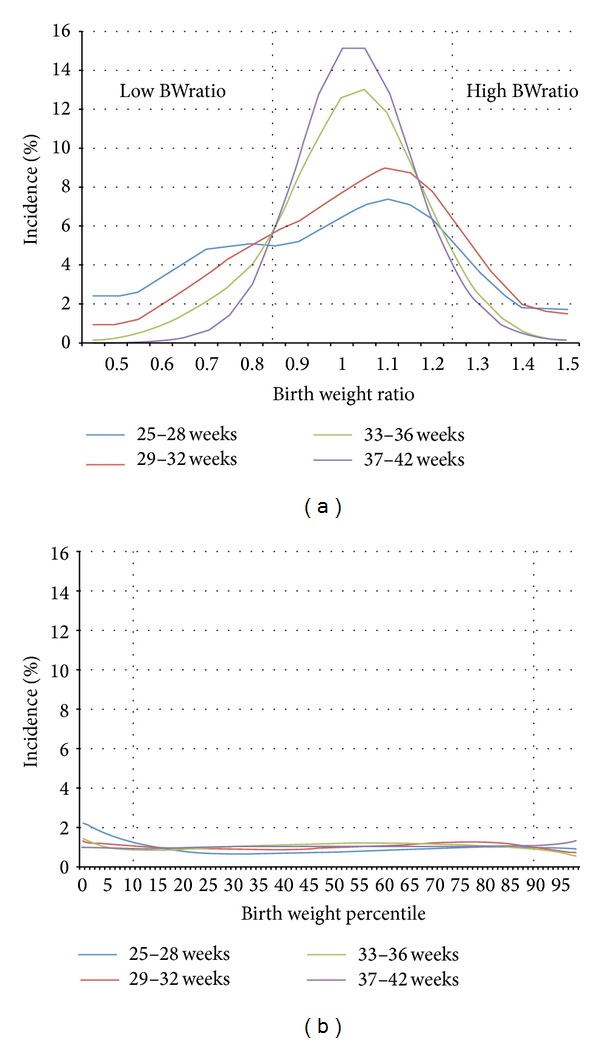
The incidence of birth weight ratios and birth weight percentiles for four strata of gestational age at birth separate for birth weight ratio (a) and birth weight percentiles (b).

The distribution of birth weight ratios and birth weight percentiles for four strata of gestational age at birth is shown in Figures 1(a) and 1(b).  Figure 1(a) shows that most infants are born with a BWratio around one and that both higher and lower BWratios are less common. Moreover, 80% of cases had a BWratio between 0.85 and 1.25. These infants occupy approximately one-third of the width of the graph, while the low BWratio and high BWratio infants occupy the remaining two-thirds. Hence, the spread in BWratio is much larger in the extremes of the birth weight ratio distribution than when using birth weight percentiles. These characteristics of BWratio distributions make it possible to better distinguish between different degrees of low BWratios and high BWratios.

Figure 1(b) contains the distribution of birth weight percentiles and shows that the population is cut into 100 (approximately) equal parts. As a result, any given percentile always contains about 1% of the population. Consequently, the central 80% of the graph represents 80% of the population, while this only corresponds to approximately one-third of the BWratio distribution. Whereas SGA and LGA infants (20% of the population) logically cover 20% of the percentile distribution, while this group represents the remaining two-thirds of the BWratio distribution.

Figure 1(a) also shows that the BWratio of late premature (33^+0^–36^+6^ weeks' gestation) and term (37^+0^–42^+6^ weeks) infants is normally distributed. Distribution of the birth weight ratio of extremely premature (25^+0^–28^+6^ weeks' gestation) or very premature infants (29^+0^–32^+6^ weeks gestation) is negatively skewed.

### 3.2. Incidence of Abnormal Growth

The lines in Figure 1(a) suggest higher rates of low BWratios and high BWratios among infants that are born preterm. At term (37–42 weeks GA), 9.67% [118,331/1,223,815] of infants are born with a BWratio < 0.85. In the preterm period the incidences are significantly higher (*P* < 0.001) than in the term group, 17.04% [10,487/61,544] (33–36 weeks), 25.73% [2,531/9,837] (29–32 weeks), and 37.8% [1,530/4,084] (25–28 weeks), respectively.

At term (37–42 weeks GA), 6.64% [81,312/1,223,815] of infants are born with a high BWratio (>1.25). In the preterm period, the incidences are significantly higher (*P* < 0.001) than in the term group, 8.43% [5,188/61,544] (33–36 weeks), 18.39% [1,809/9,837] (29–32 weeks) and 16.48% [667/4,084] (25–28 weeks), respectively.

These findings confirm the presence of an association between prematurity and the incidence of abnormal growth (BWratio < 0.85 as well as BWratio > 1.25) [13–15].

### 3.3. Perinatal Death and Composite Morbidity

**Figure 2:**
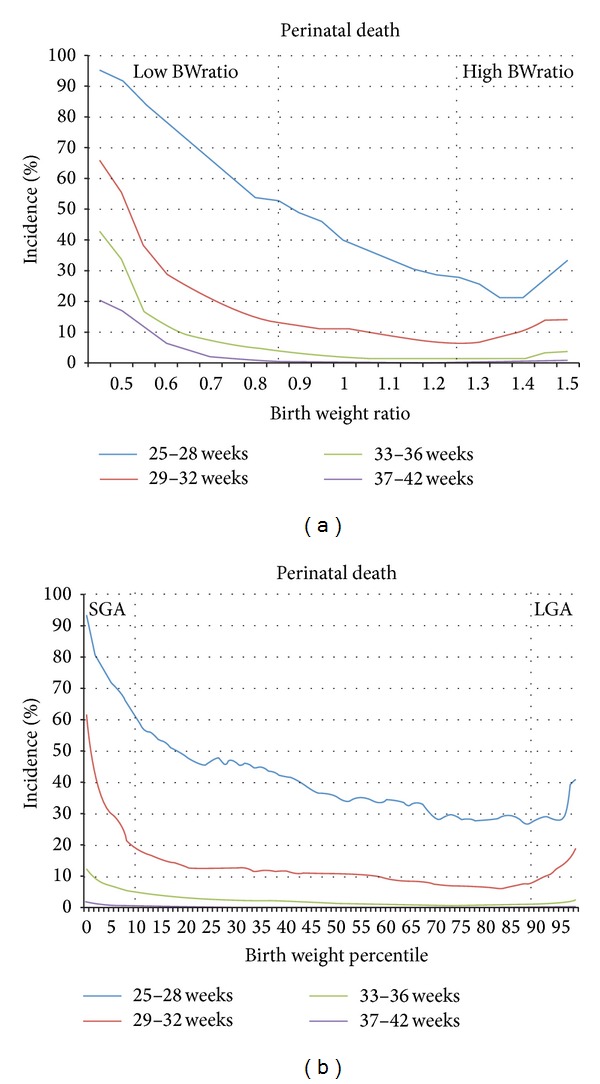
Incidences of perinatal death stratified by gestational age at delivery separate for birth weight ratio (a) and birth weight percentiles (b).

**Figure 3:**
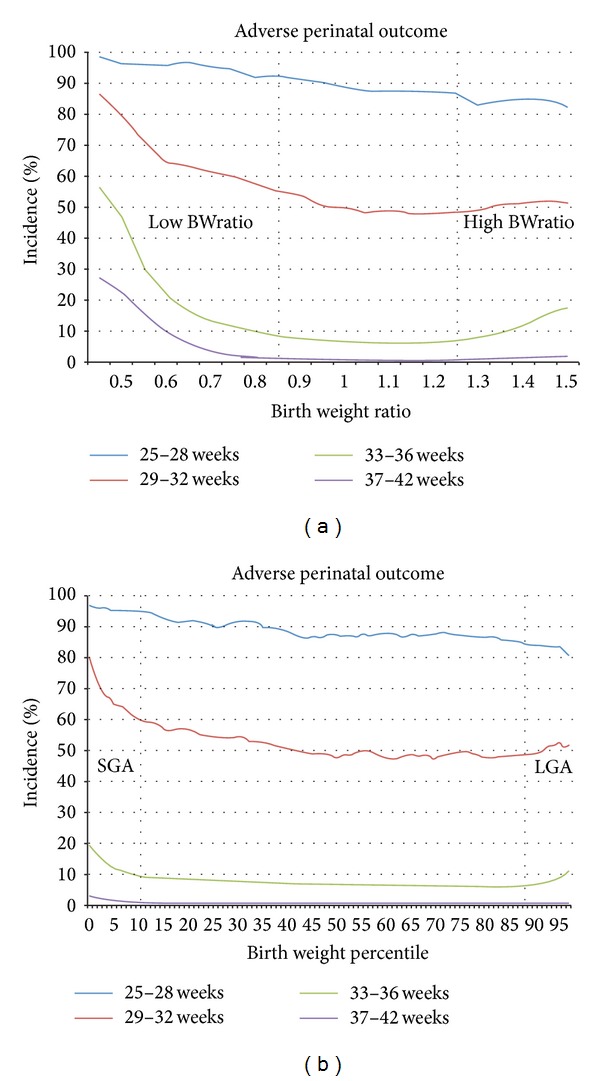
Incidences of adverse perinatal outcome stratified by gestational age at delivery separate for birth weight ratio (a) and birth weight percentiles (b).

Incidences of perinatal death and adverse pregnancy outcome are shown in Figures 2 and 3. Incidences are shown separate for four strata of gestational age at birth, by birth weight ratios (Figures 2(a) and 3(a)), and birth weight percentiles (Figures 2(b) and 3(b)).

Comparison of mortality rates in Figures 2(a) and 2(b) shows that especially in the late preterm period (33–36 weeks) and at term (37–42 weeks) birth weight ratio allows more accurate differentiation between different SGA grades than birth weight percentiles. This is illustrated by Figure 2. Although it seems in Figure 2(b) that perinatal death between 33 and 36 weeks gestation does not rise above 10% in infants with a birth weight at the 1st percentile, Figure 2(a) shows that perinatal death rate rises until over 40%, depending on the severity of growth restriction.

On the other side of the growth spectrum, birth weight ratio also allows more precise differentiation between different severities of LGA.

Both birth weight ratio and birth weight percentile show a gestation related death rate in the normal range (BWratio 0.85–1.25 and p10–p90, resp.) with higher death rates towards both ends of the growth spectrum. The same effects at the ends of the growth spectrum were found for adverse pregnancy outcome (Figures 3(a) and 3(b)).

### 3.4. Population-Attributive Risk of Abnormal Growth for Death and Adverse Pregnancy Outcome

The percentage of perinatal death and adverse pregnancy outcome that can be attributed to abnormal growth depends on gestational age at delivery, on whether abnormal growth is defined by BWratio (low/high BWratio) or BWpercentile (SGA/LGA), and on the cut-off value that is used. PAR of abnormal fetal growth for perinatal death at different gestational ages is shown in Table 2. Depending on gestation and on the definition of abnormal growth, 14–35% of perinatal death and 2–13% of adverse pregnancy outcome can be attributed to abnormal growth.

The population-attributive risk of abnormal growth is higher for death than for adverse pregnancy outcome, which means that a larger percentage of perinatal deaths than adverse pregnancy outcome can be attributed to abnormal growth. PAR of suboptimal growth for perinatal death is small in extremely premature infants, increases with advancing gestational age with a peak in late preterm infants (33–36 weeks), and decrease at term.

Also, PAR of abnormal growth, for example, for perinatal death at term, is higher if less stringent cut-off values to define abnormal growth are chosen (e.g., the 10th percentile instead of the 5th percentile, 22% versus 17%).

### 3.5. Discriminative Ability of Birth Weight Ratio and Birth Weight Percentile

The areas under the receiver operator characteristics curves are shown in Table 3. When assessing the complete growth spectrum, there were no differences in areas under the curve (AUC) between birth weight ratio and birth weight percentile to distinguish between those with and without perinatal death in extremely preterm, very preterm, late preterm, and term infants. Accordingly, the discriminative ability of birth weight ratio and birth weight percentile for our composite adverse pregnancy outcome did not differ at any gestational age either. The discriminative ability of both methods for death was poor to fair (range 0.64–0.73), and the discriminative ability for adverse pregnancy outcome was bad to poor (range 0.55–0.65) [16].

When we assessed SGA cases only (birth weight below the 10th percentile), the discriminative ability of BWratio was better than that of BWpercentile for death in the late preterm period (33^+0^–36^+6^ weeks) (0.68 versus 0.63, *P* 0.01) and at term (0.69 versus 0.67, *P* 0.05) (Table 4). The discriminative ability of BWratio was also better than that of BWpercentile for adverse pregnancy outcome in the late preterm period (33^+0^–36^+6^ weeks) (0.67 versus 0.60, *P* < 0.001).

## 4. Discussion

Discriminative ability of BWratio for perinatal death or adverse pregnancy outcome is comparable to that of birth weight percentile. Birth weight ratio is—for smaller and larger than average infants—a more discriminative instrument for perinatal death and adverse pregnancy outcome than birth weight percentile.

Our findings confirm an association between abnormal fetal growth and premature delivery [13–15], and our data show that approximately one out of five perinatal deaths can be attributed to being SGA.

### 4.1. Limitations

Some limitations need to be addressed. First, a possible limitation is related to the use population-based birth weight percentiles. Although there exists no unanimity about the question whether references should be based on population birth weight characteristics or on individual growth potential, the latter might have better discriminative ability for adverse outcome [13, 17–23], both with BWratios and BWpercentiles. We were not able to use customized growth curves because of maternal length and weight, and placental weight and pathology are not registered in the Dutch Perinatal Registry. Therefore the Dutch reference curves for birth weight by gestational age separate for parity, sex, and ethnic background were used [9]. We think however that not being able to use customized growth curves was only a minor limitation, because the concepts put forward in this paper can also be applied if growth is expressed using customized charts.

A second potential limitation is the use of preterm birth weight as standards for preterm BWratio. This might have led to an underestimation of the effect of prematurity on pregnancy outcome because—as this study shows—prematurity is associated with abnormal growth.

Finally, the PRN database does not contain data on how pregnancy dating is performed. Until 2011, no uniform pregnancy dating was performed in The Netherlands. Historically, it was common practice to date pregnancies based on LMP. Since the 1980's the use of ultrasound was gradually introduced in obstetric care. During our study period crown rump length and head circumference measurements had already increasingly replaced LMP for dating, but no quantitative data are available on how pregnancy was dated in individual cases. Inaccurate pregnancy dating might be partially responsible for the wider birth weight spread in the preterm period. However, this wider spread in the preterm period might also be the result of higher incidences of pathologically small or large growth, leading to preterm delivery.

### 4.2. Strengths

The main strength of this study is the size (1,299,244 pregnancies) and composition (only white women with a singleton without congenital anomalies) of the cohort. The incidence of fetal deaths, neonatal deaths, perinatal deaths, and composite morbidity that we found in this study is in accordance with previous research [24–29]. There is no reason to suspect a systematical gender or parity based bias. The concepts discussed in this paper can be used to assess distribution of growth and risk of abnormal growth and its relation to pregnancy outcome in other populations.

Data are derived from a large, well-maintained population-based national perinatal registry (1999–2007). The vast majority of the caregivers contribute to the PRN registry; therefore, it comprises approximately 96% of all pregnancy and birth characteristics in The Netherlands. The 4% missing birth data are due to 1-2% nonreporting general practitioners and 2-3% nonreporting midwives. The use of population-based growth curves for white mothers, separate for gender and parity, minimizes risk of systematic bias caused by one of these factors.

Finally, this is to our knowledge the first study that compared birth weight ratio and birth weight percentiles using ROC curves. This allowed us to statistically substantiate our findings.

### 4.3. Interpretation of the Results

As shown in Figure 1(b), only a distribution based on BWratio tells us how growth is distributed within a population. It shows that most infants are born with a birth weight ratio of around one, and incidences of BWratios decrease towards both ends of the distribution.

This study confirms that preterm delivery is associated with increased SGA and LGA rates as compared to term delivery [14, 15] and that the relation between fetal growth and the risk of adverse pregnancy outcome also depends on gestational age at delivery. This means that the relative risk of adverse outcome for an infant with a certain BWratio or BWpercentile at 30 weeks gestation is not the same as that at 40 weeks' gestation.

The results also show that birth weight ratios are not normally distributed in infants that are delivered extremely—or very preterm (25^+0^–32^+6^ weeks) (Figure 1(a)). Percentiles are only suitable for use in a normally distributed population and are therefore less suitable to express growth in premature and extremely premature infants.

Finally, birth weight ratio allows comparison of groups with different average weights and weight distributions, for example, male infants and female infants or infants of different ethnic origins. Percentiles and ratios both allow comparison of groups, but—as explained before—information on distance from the mean and the incidence of different ratios within a population is lost when percentiles are used.

The different representation of growth with BWratio and BWpercentile that is explained above has two effects that result in better interpretability of infant growth when BWratio is used.

First, the use of BWpercentile causes a loss of discriminative power, especially among SGA and LGA infants. For example, all infants with birthweight <1st percentile (1% of the population and BWpercentile distribution) cover about 10% of the BWratio distribution, thus allowing better differentiation within this group of small infants with BWratio.

Second, birth weight percentiles suggest that an infant (born at term) with a birth weight at the 25th percentile is much lighter than an infant at the 75th percentile. However, this is not the case. The birth weight ratios in this example are 0.9 (25th percentile) and 1.1 (75th percentile) and are both very close to 1.0. The seemingly large difference if growth is expressed in percentiles is caused by the fact that a population is by definition divided into 100 equally large groups instead of groups based on birth weight in relation to the median and that most infants have a birth weight close to the median.

There are two reasons for the fact that neither BWpercentile nor BWratio has high sensitivity and specificity for death and adverse outcome and that only a limited proportion of death and adverse outcome can be attributed to fetal growth below the 10th percentile.

First, unlike other tests, increased risk of death (or adverse outcome) is not associated with a one-directional change in the risk factor (birth weight ratio or percentile). Both low and high birth weight ratios (or percentiles) are associated with increased risk of death (or adverse outcome).

Second, both death and adverse outcome occur at all gestational ages and across the whole growth spectrum. There seems to be a gestational age related basic risk (horizontal part of the line) with increased death and adverse outcome rates at both ends of the growth spectrum.

## 5. Conclusions

In view of the results we think that BWratio could complement BWpercentiles in clinical practice and can play a role in scientific research. It allows differentiation of SGA infants that is not possible with BWpercentile; it is easy to understand and therefore useful for patient counseling. This study provides in our opinion sufficient evidence for clinicians to use birth weight ratios when assessing risks of adverse outcome when an infant is suspected to be extremely small or large for gestation. Finally, birth weight ratio enables comparison of populations with different baseline characteristics.

This study shows the need to redefine cut-off values that define abnormal fetal growth. Historically, these cut-off values have been set at the 2.5th, 5th, and 10th percentile. However, this study shows that the relation between fetal growth and the risk of adverse pregnancy outcome differs depending on gestational age at delivery. Therefore, future research should focus on defining cut-off values to identify infants at risk of clinically relevant poor growth. To do this, consequences of abnormal growth should be weighed against potential treatment benefit of early detection and intervention, also taking into account costs of follow-up and potential adverse effects of interventions. Given the potentially better associations with adverse pregnancy outcome, such research should be performed using customized weight percentiles.

## Figures and Tables

**Table 1:** Characteristics of the 1,299,244 pregnancies in the Netherlands, 1999–2007.

	Total
	(*n* = 1,299,244)
Maternal characteristics	
Maternal age, mean, (SD)	30.7 (4.58)
Nulliparous, %	47.5
Low socioeconomic status, %	18.9
Boys	51.3
Pregnancy and delivery	
Induction of labor, %	35.3
Cesarean section	14.3
Elective cesarean section %	6.2
Emergency cesarean section %	11.3
Vaginal instrumental delivery	12.4
Neonatal characteristics	
Gestational age at delivery (weeks), median (IQR)	39,2 (1.86)
Extremely premature (GA < 29^+0^ weeks), *n* (%)	4,048 (0.3)
Very premature (GA < 33^+0^ weeks), *n* (%)	13,885 (1.1)
Mild premature (GA < 37^+0^ weeks), *n* (%)	75,429 (5.81)

SD, standard deviation.

**Table 2:** Population-attributive risk of abnormal fetal growth, for four definitions of abnormal growth by birth weight ratio and birth weight percentile.

	Population-attributive risk percentage (PAR%)			
	Birth weight ratio		Birth weight percentile	
	<0.80	<0.85	<p5	<p10
25–28 weeks				
Perinatal death	26	28	18	23
Composite adverse outcome	3	3	2	2
29–32 weeks				
Perinatal death	24	27	14	20
Composite adverse outcome	5	6	3	4
33–36 weeks				
Perinatal death	29	35	19	25
Composite adverse outcome	11	12	10	13
37–42 weeks				
Perinatal death	18	24	17	22
Composite adverse outcome	9	11	7	8

**Table 3:** Discriminative ability to predict perinatal death or adverse outcome of birth weight ratio and birth weight percentile.

	Area under the curve		*P* value
	Birth weight ratio	Birth weight percentile	
25–28 weeks			
Perinatal death	0.73	0.73	0.78
Composite adverse outcome	0.65	0.65	1.00
29–32 weeks			
Perinatal death	0.65	0.65	0.88
Composite adverse outcome	0.56	0.56	0.82
33–36 weeks			
Perinatal death	0.70	0.69	0.69
Composite adverse outcome	0.56	0.56	1.00
37–42 weeks			
Perinatal death	0.64	0.64	0.77
Composite adverse outcome	0.55	0.55	0.22

**Table 4:** Discriminative ability of birth weight ratio and birth weight percentile in case of birth weight below the 10th percentile for gestational age.

	Area under the curve		*P* value
	Birth weight ratio	Birth weight percentile	
25–28 weeks			
Perinatal death	0.70	0.76	0.09
Composite adverse outcome	0.61	0.63	0.75
29–32 weeks			
Perinatal death	0.69	0.68	0.67
Composite adverse outcome	0.61	0.58	0.43
33–36 weeks			
Perinatal death	0.68	0.63	**0.01**
Composite adverse outcome	0.67	0.60	**<0.001**
37–42 weeks			
Perinatal death	0.69	0.67	0.05
Composite adverse outcome	0.65	0.64	0.15
